# Awakening the control of the ankle dorsiflexors in the post-stroke hemiplegic subject to improve walking activity and social participation: the WAKE (Walking Ankle isoKinetic Exercise) randomised, controlled trial

**DOI:** 10.1186/s13063-022-06545-w

**Published:** 2022-08-16

**Authors:** Béatrice Ferry, Maxence Compagnat, Jules Yonneau, Laurent Bensoussan, Geoffroy Moucheboeuf, François Muller, Bertrand Laborde, Anne Jossart, Romain David, Julien Magne, Loïc Marais, Jean-Christophe Daviet

**Affiliations:** 1 Laboratoire HAVAE (UR 20217), EA6310, Université de Limoges, Limoges, France; 2 Médecine Physique et de Réadaptation, CHU Limoges, Limoges, France; 3 Institut Universitaire de Réadaptation, Marseille, France; 4 Médecine Physique et Réadaptation, AP-HM, Marseille, France; 5 Médecine Physique et Réadaptation, CHU Bordeaux, Bordeaux, France; 6 Médecine Physique et Réadaptation, Centre Rééducation Les Embruns, Bidart, France; 7 Médecine Physique et Réadaptation, CHU Poitiers, Poitiers, France; 8 Centre d’Epidémiologie de Biostatistiques et Méthodologie de la Recherche, CHU Limoges, Limoges, France; 9 Direction de la Recherche et de l’Innovation, CHU Limoges, Limoges, France

**Keywords:** Stroke, Rehabilitation, Ankle, Gait, Isokinetic, Sub-acute, Social participation

## Abstract

**Background:**

Stroke is the leading cause of acquired disability in France. While 90% of patients recover the ability to walk, it is often limited with a steady speed of approximately 0.7 m/s. This limitation of walking activity is partly related to a decrease in strength associated with more or less significant spasticity. In particular, it seems that the strength of the dorsiflexor muscles is directly related to walking speed. We hypothesise that a protocol based on gestural repetition targeted at the ankle during the subacute phase potentiates the recovery of motor control, improving walking activity, and participates in recovering better social participation.

**Methods:**

An estimated total of 60 patients with subacute stroke will be recruited to participate in this multicentre, interventional, prospective, randomised controlled trial.

All participants will benefit from conventional rehabilitation. In addition, the experimental group will take part in an ankle isokinetic rehabilitation programme for 6 weeks (at least 25 sessions). The control group will receive the same duration of conventional rehabilitation.

The primary outcome measure will be a 10-m walking speed at post-intervention. Secondary outcomes will include social participation, walking spatio-temporal parameters, and dorsiflexor strength. Outcome measurements will be taken at baseline, immediately after treatment (6 weeks), then at 6 months and 1 year of follow-up.

**Discussion:**

This study aims to provide scientific evidence that a protocol based on an early over-solicitation of the ankle dorsiflexor muscles to promote their “awakening” can serve to achieve a more effective walking activity, which in turn encourages social participation following discharge from the hospital.

This protocol should also help optimise physical medicine and rehabilitation practices: the more systematic use of the isokinetic dynamometer as a technique associated with, and integrated into the conventional rehabilitation protocol would allow an objective evaluation of the rehabilitation benefits and should increase the rehabilitation gain in central nervous system disorders.

**Trial registration:**

Limoges University Hospital is the sponsor of this research (Unique Protocol ID: 87RI18_0010)

This research is supported by the French Ministry of Health (PHRC 2020-A03328-31) and is conducted with the support of DGOS (PHRC interregional – GIRCI SOHO).

The study protocol was approved by the French Human Subjects Protection Review Board (Comité de Protection des Personnes Nord-Ouest III) on February 23, 2021.

The trial was registered in the ClinicalTrials.gov registry (NCT04800601) on March 16, 2021.

## Administrative information

Note: the numbers in curly brackets in this protocol refer to SPIRIT checklist item numbers. The order of the items has been modified to group similar items (see http://www.equator-network.org/reporting-guidelines/spirit-2013-statement-defining-standard-protocol-items-for-clinical-trials/).

**Table:** 

Title {1}	Awakening of the Control of the Ankle Dorsiflexors in the Post Stroke Hemiplegic Subject to Improve Walking Activity and Social Participation: the WAKE (Walking Ankle isoKinetic Exercise) randomised, controlled trial.
Trial registration {2a and 2b}.	Limoges University Hospital is the sponsor of this research (Unique Protocol ID: 87RI18_0010). The study protocol was approved by the French Human Subjects Protection Review Board (Comité de Protection des Personnes Nord-Ouest III) on 23 February 2021 (Approval number: 20.12.17.41627). The trial was registered in the ClinicalTrials.gov registry (NCT04800601) on 16 March 2021.
Protocol version {3}	Version no.2 of 16 February 2021
Funding {4}	This research is supported by the French Ministry of Health (PHRC 2020-A03328-31) and is conducted with the support of DGOS (PHRC interregional – GIRCI SOHO).
Author details {5a}	Béatrice Ferry^1^, Maxence Compagnat^1,2^, Jules Yonneau^2^, Laurent Bensoussan^3,4^, Geoffroy Moucheboeuf^5^, François Muller^6^, Bertrand Laborde^6^, Anne Jossart^7^, Romain David^7^, Julien Magne^8^, Loïc Marais^9^ and Jean-Christophe Daviet^1,2^* **Affiliations:** * Correspondence: jean-christophe.daviet@unilim.fr ^1^ Laboratoire HAVAE (UR 20217), EA6310, Université de Limoges, Limoges, France, ^2^ Médecine Physique et de Réadaptation, CHU Limoges, France, ^3^ Institut Universitaire de Réadaptation, Marseille, France ^4^ Médecine Physique et Réadaptation, AP-HM, Marseille, France. ^5^ Médecine Physique et Réadaptation, CHU Bordeaux, France. ^6^ Médecine Physique et Réadaptation, Centre Rééducation Les Embruns, Bidart, France. ^7^ Médecine Physique et Réadaptation, CHU Poitiers, France. ^8^ Centre d’Epidémiologie de Biostatistiques et Méthodologie de la Recherche, CHU Limoges, France. ^9^ Direction de la Recherche et de l’Innovation, CHU Limoges, France
Name and contact information for the trial sponsor {5b}	Limoges University Hospital, 2 avenue Martin Luther King 87042 Limoges Cedex
Role of sponsor {5c}	The sponsor has taken the initiative for a clinical trial and assumes the responsibilities and funding for this latter. In practice, the sponsor is responsible for the entire organisation, implementation and monitoring of the clinical trial: choosing the investigator, ensuring data management and carrying out quality controls, obtaining the favourable opinion of the human subjects’ protection review board and the authorisation of the competent authority, taking out insurance and declaring to the competent authority any undesirable events that occur during the research.

## Introduction

### Background and rationale {6a}

Stroke management is a real public health issue in view of the frequency, the mortality rate and the risk of disability for victims. The main post-stroke disability is hemiplegia. When it affects the lower limbs, it often results in a loss of muscle strength associated with varying degrees of spasticity. Walking activity is then impacted. Kwong et al. [1] and Faria-Fortini et al. [2] define walking activity as the best predictor and the most related to social participation. Thus, if it is not fully recovered, the individual's reintegration into society can be problematic, leading to a decrease in quality of life and social participation.

In a recent systematic review, Mentiplay et al. [3] confirmed that muscle weakness is a major contributor to decreased motor performance, in particular walking speed. Specifically, this review shows that the strength of the dorsal ankle flexor muscles is most strongly correlated with walking activity compared to other lower limb muscle groups. Kwong et al. [1], in a more recent study, confirmed these findings. They showed that, more than the other muscle groups assessed (plantar flexor muscles, knee flexor and extensor muscles), dorsiflexion strength is clearly related to walking activity. Most studies, however, have focused on strengthening knee or hip muscles. Only a few studies (randomised controlled) have studied the effects of ankle-specific management on walking activity (May et al. [4], Hsieh [5], Park et al. [6], Yoo et al. [7], An and Won [8], Rydwik et al. [9]). With the exception of the May et al. study, patients in these studies had a long post-stroke delay (8 to 62 months).

It is well known that, given the central origin of motor impairment, neuroplasticity mechanisms play a central role in stroke recovery [10]. Gestural repetitions can enhance this mechanism. Thus, task-oriented therapies such as treadmill exercises can improve gait velocity after a stroke [11]. As slow velocities and abnormal gait patterns often persist, however, Forrester et al. [12] suggest a need for additional strategies to improve walking.

According to the Cochrane review by Pollock et al. [13], motor rehabilitation should be started early and practiced with sessions of 30 to 60 min, 5 to 7 days a week.

In this context, we hypothesise that a protocol based on gestural repetition targeted on the ankle during the subacute phase, potentiates the recovery of motor control, improving walking activity, and participates in recovering better social participation.

### Objectives {7}

The primary objective is to evaluate the impact of an analytical and repetitive ankle mobilisation programme (combined with conventional rehabilitation including repetitive gait training) on comfortable walking speed over 10 m at the end of the intervention.

The secondary objectives are (1) to evaluate the impact of the programme on social participation at 6 months and 1 year, and to measure its evolution, (2) to evaluate the impact of the programme on the spatiotemporal parameters of walking at the end of the intervention and to measure their evolution at 6 months and 1 year, (3) to evaluate the impact of the programme on the progression of walking speed at 6 months and 1 year, (4) to evaluate the impact of the programme on dorsiflexor muscle strength and to measure its progression over time, at baseline and just after the intervention, along with at 6 months and 1 year of follow-up, (5) to evaluate the impact of the programme on the use of technical aids and on the number of falls between 6 months and 1 year, and (6) to evaluate the relationship between dorsiflexor muscle strength, walking speed and social participation.

### Trial design {8}

We plan an experimental, multicentre, parallel-group, randomised, controlled trial (1:1): control group vs. experimental group.

## Methods: Participants, interventions and outcomes

### Study setting {9}

The RCT (Randomised Controlled Trial) will be conducted at five centres (physical medicine and rehabilitation departments of Bordeaux, Limoges, Marseille, Poitiers and Bidard). Participants will be recruited from each respective inpatient stroke rehabilitation unit over a period of eighteen months.

### Eligibility criteria {10}

The study is presented to all patients hospitalised in the physical medicine and rehabilitation departments for a first stroke and meeting all other eligibility criteria: (1) they are within 15 days to 3 months of stroke onset (ischaemic or haemorrhagic); (2) they have persistent foot lift deficiency (Medical Research Council testing: MRC<5); (3) they are able to walk alone at least 10 m with or without technical assistance; (4) they do not have pain in the paretic lower limb (AVS<2); and (5) they are cleared to participate in physical therapy.

Patients will be excluded from the study if they (1) have cognitive or phasic disorders preventing them from understanding the instructions: Boston Scale BDAE<2; (2) had gait disorder before the stroke; (3) have fixed contracture of the ankle (irreducible equinus); (4) are too spastic: Modified Ashworth (MAS) greater than or equal to 4; (5) are pregnant, have a desire for pregnancy or are breastfeeding; and (6) are under curatorship or guardianship or under the protection of justice.

### Who will take informed consent? {26a}

The investigator will inform the patient. Patients will be enrolled after providing written informed consent. The informed consent is signed by the patient and the investigator.

### Additional consent provisions for collection and use of participant data and biological specimens {26b}

If a patient withdraws consent, their data will be removed from the database and not used for analyses. In the event of study discontinuation, the data may be retained if authorised by the patient. In such cases, primary and secondary outcomes will be evaluated at the date of discontinuation.

### Interventions

#### Explanation for the choice of comparators {6b}

We wish to compare a new rehabilitation technique to conventional rehabilitation. The control group is therefore the one with conventional treatment (the two groups have the same treatment duration).

#### Intervention description {11a}

All patients will receive conventional physiotherapy rehabilitation for 1 h per day, 5 days a week. This conventional management will be performed at the same location, under the guidance of the same physical therapist team to ensure consistent performance of the protocol and safety of all enrolled patients. Conventional rehabilitation includes repeated active and/or passive mobilisations, stretching and postures to inhibit associated spasticity, neurofacilitation techniques, sensory-motor rehabilitation. As soon as possible, patients will be put on their feet, upright with work on balance and weight transfer associated with walking training, repetitive, with technical assistance if necessary. Finally, a fall prevention programme will be implemented. Both groups receive the same amount of rehabilitation.

1. Experimental group (EG): the experimental procedure will consist of analytical retraining of the ankle on an isokinetic dynamometer to awaken motor control and promote strength gain. It will consist of an additional 30 min of isokinetic ankle rehabilitation. The patient is installed on the seat of the isokinetic dynamometer. The trunk-thigh (hip) angle is set at 85°, the thigh-leg (knee) angle at 50° and the foot-leg (ankle) angle at 90°. In order to stabilise the position, the trunk, thigh and foot of the tested leg are strapped. The joint range for plantar flexion/dorsiflexion movement is determined for each subject, subjectively based on how each individual feels. The dynamometer performs automatic gravity correction.

Analytical ankle retraining consists in performing a series of plantar flexion/dorsiflexion movements in CPM (Continuous Passive Mobilisation) mode. The instruction given to the patient will be to accompany the movement by pressing (plantar flexion) or pulling (dorsiflexion) with their foot as hard and as fast as possible, with the intention of achieving maximum active movement. In this way, patients without control are in passive mobilisation but with the intention of achieving voluntary movement. Patients with low motor skills are in assisted active movement and patients with sufficient control are in concentric contraction. The CPM training modality allows subjects to work regardless of the level of recovery in order to reactivate analytical motor control of the ankle.

Among the protocols already published with stroke patients in the acute phase, Vér et al. [14] proposed 210 passive movements in the acute phase in 30 min, 5 times per week. With chronic patients, Forrester et al. [12] proposed 720 active movements in 1 h 3 times per week. The duration of the protocols is most often 5 (An et Won [8], An et Jo [15]) to 6 weeks (Forrester et al. [12, 16]).

From these data, we propose a re-training of 300 movements in 30 min, 5 times a week for 6 weeks, with a minimum of 25 sessions. The protocol comprises 6 sets of 50 repetitions at 30°/s (2min 45 per set) interspersed with 2 min of rest, i.e. a session lasting a total of 28min 30.

2- Control group (CG): the comparative procedure consists of a conventional rehabilitation programme as described before. Patients in CG will have a supplement in conventional rehabilitation equal to the additional time of the experimental group (30min).

### Criteria for discontinuing or modifying allocated interventions {11b}

N/A; no criteria for interruption or modification of allocated interventions.

### Strategies to improve adherence to interventions {11c}

N/A; no strategies to improve adherence to interventions.

### Relevant concomitant care permitted or prohibited during the trial {11d}

Other rehabilitation modalities necessary for the patient are allowed (occupational therapy, speech therapy, neuropsychology) and drug treatments are allowed.

No procedure is forbidden, except for the introduction of additional physiotherapy sessions that could lead to an imbalance in the amount of rehabilitation between the 2 groups.

### Provisions for post-trial care {30}

N/A; no provisions for post-trial care.

### Outcomes {12}

All testing sessions will be conducted by one physical therapist and one research assistant (per centre). Both evaluators will be blinded to the treatment.

**Fig. 1:**
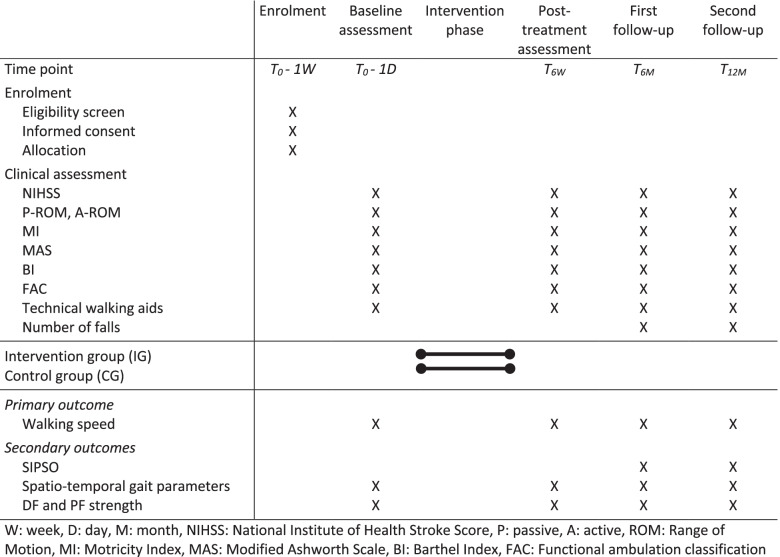
Schedule of enrolment, intervention, assessment and follow-up

Clinical and physical outcome measurements will be administered to the patients at baseline, immediately after 6 weeks of treatment, 6 months and 1 year after treatment (Fig. 1).

The *primary outcome* is defined as walking speed measured over 10 m at the end of the intervention (6 weeks).

The *secondary outcome* includes social participation evaluated at 6 months and 1 year, walking spatiotemporal parameters measured at each visit, walking speed measured at 6 months and 1 year, along with dorsiflexor strength evaluated at each visit.

The use of technical aids to walk and the number of falls will be described.

Finally, correlations between walking speed/dorsiflexor muscle strength and walking speed/social participation will be studied.

#### Outcome measurements

*Walking speed* will be evaluated by a 10-m walking test (10MWT). The 10MWT is a functional evaluation tool that can be easily used in clinical practice. The participants will walk this distance at a comfortable speed. Considering the initial acceleration and the last deceleration, the patients start walking 2 m before and stop 2 m after the start of the measurement. The walking time for a 10-m distance will be repeatedly measured three times, and the mean value will be selected for analysis. The verbal instruction for walking at a comfortable pace is, “If I say start, walk at a comfortable pace until I say to stop”. The test-retest reliability of this method is well-documented in post-stroke patients with a good intra-class correlation coefficient (ICC) of 0.83 for Chen et al. [17] and 0.86 for Fulk et Echternach [18].

*Walking spatiotemporal parameters* will be recorded on a GAITRite mat (GAITRite Platinum; CIR Systems, USA). The electronic treadmill (7.01 m long by 0.61 m wide) used during this study consists of sensors arranged in the form of a grid (48 × 24 sensors) for a resolution of 1.27 cm × 1.27 cm. The acquisition frequency is set at 80 Hz. We will employ 4 parameters: durations of stance and swing phases, cadence and step variability. In addition to its ease of use and reliability, this system has many advantages, such as the possibility of walking with technical aids (walkers, canes). Three passages will be carried out and the average of the 3 passages will be kept and then studied. The experimenter explains each task before it is carried out and gives an example to ensure that the patient understands it correctly. The test-retest reliability of this system for measurement of spatiotemporal gait parameters was very good (ranging from 0.98 to 0.99) in post-stroke patients [19].

*Ankle muscle strength:* Dorsiflexor muscle strength will be measured with an isokinetic dynamometer (CON-TREX, Physiomed). The patient is installed on the seat of the isokinetic dynamometer. The trunk-thigh (hip) angle is set at 85°, the thigh-leg (knee) angle at 50° and the foot-leg (ankle) angle at 90°. In order to stabilise the position, the trunk, the thigh of the tested leg and the foot are strapped. The joint range for plantar flexion/dorsiflexion movement is determined for each subject, subjectively, based on how each individual feels. The dynamometer performs automatic gravity correction. The test will be carried out in concentric/concentric mode at a speed of 30°/s. Before the test, the patient will perform submaximal repetitions to warm up and for habituation. The test will consist of 3 maximum flexion/extension petitions. The data collected will be the maximum dorsiflexor peak torque. According to the systematic review by Rabelo et al. [20], the isokinetic dynamometer seems to display good reliability for use in post-stroke patients with an ICC greater than 0.8, although fewer studies have been conducted on the ankle.

*Social participation* will be evaluated with the Subjective Index of Physical and Social Outcome (SIPSO) at 6 months and at 1 year. SIPSO is a 10-question social participation questionnaire specially developed for stroke [21, 22] with a scale of 0 to 5 divided into two subscales: 5 questions relating to physical function (physical component) and 5 questions concerning the social and emotional domains (social component). The test-retest reliability of SIPSO is excellent (ICC = 0.96) and shows high validity in stroke populations [21]. Higher scores on SIPSO reflect higher levels of ambulation, mobility, leisure, social interaction and communication, along with a high level of reintegration.

### Participant timeline {13}

#### Inclusion

The inclusion visit will be conducted by the physician within 1 week of the pre-inclusion visit. It is carried out during hospitalisation, between 15 days and 3 months post-stroke. The investigator will perform a clinical examination to verify the inclusion and non-inclusion criteria. Once the criteria have been verified, the patient and investigator will sign the informed consent form.

The investigator will collect data on the patient's stroke pathology.

The clinical examination will then be completed to provide clinical scores: severity of the stroke (National Institute of Health Stroke Score; NIHSS), passive and active range of motion of the ankle (P-ROM, A-ROM: manual measurement), motor control of the injured lower limb (De Meurisse Motor Index; MI), assessment of spasticity of the posterior leg compartment (Modified Ashworth Scale; MAS), level of functional independence (Barthel index; BI) and walking autonomy (Functional Ambulation Classification; FAC) with collection of the technical aids used and the number of falls.

This clinical evaluation will be completed by instrumental evaluations: walking speed, spatiotemporal parameters and dorsiflexor muscle strength.

#### Randomisation

Eligible patients who agree to participate will be asked to give their written informed consent, and will then be randomised into the EG or CG. We will randomise the patients at the first clinical examination. The randomisation process will be ensured using a secure internet connection with access by individual user ID and password and encrypted data transmission to the randomisation platform of the Clinical Research Unit of the Limoges University Hospital. Only the study methodologist will complete and keep the randomisation list on a secure server. In the event of a computer problem, a paper randomisation list will be available. This randomisation list will not include a stratification factor. All actions carried out on this platform, along with the identity of the person who made the changes, will be automatically archived by audit trail procedure. As soon as a patient is randomised, an email will automatically be sent to all study organisers, i.e. the clinical research assistant, data manager and principal investigator. No strategy is provided for preventing a potential imbalance between intervention and control group allocation.

#### Follow-up

Regardless of the group, patients will benefit from a 1-year follow-up including 4 visits: the first and second visits will take place during patient hospitalisation. The third (at 6 months) and the fourth (at 1 year) visits will be performed as part of the patient’s usual follow-up in the physical medicine and rehabilitation centres.

At each of these visits, a doctor will perform a clinical examination, a physical therapist and a research assistant will perform the tests to obtain the necessary outcomes for the study.

### Sample size {14}

We calculated the number of subjects in reference to the study by Gharib et Rabab, 2017 [23]. The average speed is 0.58+/−0.09 m/s in the control group and 0.67+/−0.11 m/s in the experimental group. The estimated difference between the 2 groups is 0.09 m/s. For a first species risk of 0.05 and a power of 90%, bilaterally, the number of subjects required is 54 subjects (27 per group) plus 10% (to compensate for those lost to follow-up or excluded from study). The number of patients to be included is therefore 60 (30 subjects per group).

### Recruitment {15}

Five centres that are used to working together will be involved in this study. A preliminary assessment of the feasibility of the project has been carried out and has shown that these centres have all the necessary equipment and expertise for this research.

### Assignment of interventions: allocation

#### Sequence generation {16a}

Eligible patients will be randomly assigned at a 1:1 ratio to the experimental or control group using a web-based randomisation system.

#### Concealment mechanism {16b}

Randomisation will be centralised using the randomisation platform of the Centre d’Epidémiologie, de Biostatistique et de Méthodologie de la Recherche of the Limoges University Hospital (CEBIMER) and will be conducted over a secure internet connection. In the event of a computer problem, a paper randomisation list will be available.

#### Implementation {16c}

When an investigator wishes to perform the randomisation after verifying the eligibility of a participant, he/she logs onto the platform website. The investigator completes the “randomisation” web page after having previously confirmed all of the eligibility criteria of the participant on the site. After validation of the content, randomisation is carried out and the site immediately communicates to the investigator the result of the randomisation, in particular the procedure group allocated to the participant.

### Assignment of interventions: Blinding

#### Who will be blinded {17a}

The design is open label: investigators and patients will be aware of group allocation with only outcome assessors being blinded. Evaluators will be blinded. They will not know whether the patient is included in EG or CG. Data analysts will be blinded.

#### Procedure for unblinding if needed {17b}

N/A: the design is open label with only outcome assessors being blinded so unblinding will not occur.

### Data collection and management

#### Plans for assessment and collection of outcomes {18a}

The investigators will collect data in the Ennov Clinical eCRF. Ennov clinical is a software application for clinical trial data management which uses the ORACLE database.

Patient data will be entered into the database by each user via an online portal. Each user will log onto the secure URL with a user name and a password which grants them specific viewing and editing rights (depending on the user’s profile).

The database will be hosted on the platform of the Centre d’Epidémiologie, de Biostatistique et de Méthodologie de la Recherche of the Limoges University Hospital (CEBIMER) in the Limoges Hospital. Data will be saved in real time and archived every day as computer files and on a protected hard-drive.

#### Plans to promote participant retention and complete follow-up {18b}

Patient follow-up data is entered into the eCRF and the end of research is documented, including reasons for premature discharge.

#### Data management {19}

Data will be managed by the CEBIMER. The data will be entered online by the investigators after logging onto the study database, which will be created by a data manager using the Ennov Clinical software. The quality of data entry will be ensured by consistency tests configured and generated by the data manager. If an element is incorrectly completed, validation messages will be sent to the investigator.

The WAKE trial will have on-site monitoring. An initial monitoring visit is planned for all participating centres at the start of the inclusion period. All centres will be visited by independent monitors according to the pace of recruitment. The monitor will check the informed consent forms, the consistency of the data entered with the source data and whether the inclusion and non-inclusion criteria were followed.

#### Confidentiality {27}

Authorised University Hospital staff and health authorities may have direct access to patients’ medical records in order to check the accuracy of the data collected. All these individuals are subject to professional secrecy. Medical records identifying the patient will be kept in the hospital and remain confidential.

#### Plans for collection, laboratory evaluation and storage of biological specimens for genetic or molecular analysis in this trial/future use {33}

N/A; no collection, laboratory evaluation or storage of biological specimens for genetic or molecular analysis.

## Statistical methods

### Statistical methods for primary and secondary outcomes {20a}

The statistical analysis will be performed by the CEBIMER (Centre d’Epidémiologie, de Biostatistique et de Méthodologie de la Recherche) of the Limoges University Hospital using SAS software V9.3 (SAS Institute, Cary, NC).

#### Primary endpoint analysis

The comparison of the mean walking speed over 10 m measured at the end of the intervention (week 6) will be done by a Student’s *t* test if the variable follows a normal distribution (Shapiro-Wilks test) or by a non-parametric Mann-Whitney test otherwise.

#### The following secondary endpoint analyses:

- The comparison of the mean difference of the SIPSO score between 6 months and 1 year (T_12M_ – T_6M_) will be performed by means of a Student's t test if the variable follows a normal distribution (Shapiro-Wilks test) or by a non-parametric Mann-Whitney test otherwise.
- The comparison of the progression of the 4 spatiotemporal parameters selected (duration of the stance and swing phases, step cadence and variability) will be performed using a mixed generalised linear regression.
- The comparison of the progression of the 2 parameters describing the force of the dorsi-flexor muscles (peak torque in Nm and active amplitude in degrees) will be performed using a generalised linear regression.
- The comparison of the proportion of patients who used a technical aid will be performed by a Pearson’s chi [2] test or a Fisher’s exact test if at least one theoretical number is <5). The comparison of the number of falls per patient after 6 months will be performed by a binomial negative regression.
- Correlations between [1] dorsi-flexor muscle strength (2 parameters) and walking speed at 6 weeks, 6 months, and 1 year [2]; dorsi-flexor muscle strength (2 parameters) and SIPSO score at 6 months and 1 year [3]; walking speed and SIPSO score at 6 months and 1 year will be performed using the Pearson or Spearman correlation coefficient test depending on the conditions of application.

### Interim analyses {21b}

N/A; no interim analyses.

### Methods for additional analyses (e.g. subgroup analyses) {20b}

N/A; no methods for additional analyses.

### Methods in analysis to handle protocol non-adherence and any statistical methods to handle missing data {20c}

If any, the frequency of missing values will be described. If relevant, according to the proportion and mechanism of the missing values, an appropriate method to replace the missing data will be used. Robustness analysis will be performed (using maximum scenarios for missing value replacement) in order to evaluate the robustness of the results.

### Plans to give access to the full protocol, participant level-data and statistical code {31c}

The project is made public on clinical trials and the results are made available to patients on request. The datasets analysed during the current study and statistical code are available from the corresponding author on reasonable request, as is the full protocol.

### Oversight and monitoring

#### Composition of the coordinating centre and trial steering committee {5d}

The coordinating centre of the Limoges University Hospital is composed of the coordinating investigator, the project manager, the methodologist, a data manager and a biostatistician

#### Composition of the data monitoring committee, its role and reporting structure {21a}

N/A. There is no data monitoring committee – Ethics committee assessed the study as a low-risk intervention. A data review meeting is planned with all members of the coordinating centre.

#### Adverse event reporting and harms {22}

In accordance with French legislation, the investigators will report to the sponsor (Limoges University Hospital), as soon as they are informed, serious adverse events (SAEs) requiring hospitalisation or resulting in death.

#### Frequency and plans for auditing trial conduct {23}

Project Management Group meet to review trial conduct three times a year.

#### Plans for communicating important protocol amendments to relevant parties (e.g. trial participants, ethical committees) {25}

Any substantial modification to the protocol requires the opinion of the ethics committee. In the event of a protocol change, the sponsor and funder will be notified first, then the principal investigator at each centre, and a copy of the revised protocol will be sent to be added to the investigator's site file. Any deviations from the protocol will be fully documented using a breach report form.

#### Dissemination plans {31a}

Any written or oral communication of the study results must have been previously agreed by the coordinating investigator. The publication of the main results should include the name of the sponsor, all investigators who recruited or followed the patients in the study, the persons who participated in the study and the source of funding. International writing and publication rules (Vancouver Agreement, February 2006) will be taken into account.

## Discussion

The direct benefits expected from this research are an increase in walking activity and greater social participation. We hope that the early, repeated, assisted solicitation of the ankle muscle, with the intention of carrying out the movement, will result in a more rapid awakening of the motor command, and therefore in greater muscular solicitation and less loss of strength.

The proposed isokinetic protocol is intended for all patients regardless of the extent of their motor deficit. Indeed, the fact of proposing a protocol in assisted mode enables early passive repetitive mobilisation for the most deficient patients, ensuring an early effect on post-injury plasticity. This mobilisation continues in an active assisted mode as soon as sufficient control reappears, which contributes to the recovery of voluntary control and strength. The improvement in motricity should have consequences on the walking activity and facilitate social participation.

Several studies have shown that repeated mobilisation of the ankle can have a beneficial effect on walking. Cho and Park [24] showed that combination therapy of joint mobilisation and active stretching improves the range of motion of the ankle joint and spatiotemporal gait variables (cadence, speed, stride length). Park et al. [6], along with An and Won [8], showed that mobilisation with movement improves ankle range of motion and gait velocity. Hsieh [5] proposed a rehabilitation programme based on the use of videogames in which plantar/dorsi flexion movement is used to control the mouse. This programme had a positive effect on walking speed and balance control. All these studies concerned chronic patients. We posit that if repetitive mobilisations are performed in the subacute phase, the benefits on walking will be more marked. Also, the main limitation of these different studies is the small size of their populations, making it difficult to generalise the results. Our study could confirm the impact of early ankle mobilisation on walking and suggest effects on social participation. Because this is a multicentre study, including a larger population, the results should be more easily generalisable.

The isokinetic rehabilitation protocol we have chosen could be discussed. We developed it from the synthesis of studies finding a positive effect of a specific protocol of repetitive ankle mobilisation as explained in the intervention section. The protocols applied in the studies preceding ours propose 210 [14] to 720 [12] movement repetitions, 3 to 5 times per week for up to 6 weeks [12, 16]. We have voluntarily chosen a programme that is sufficiently intensive and prolonged to offer a chance of observing a positive effect. From these data, we propose a retraining of 300 movements in 30min, 5 times a week for 6 weeks, with a minimum of 25 sessions.

One limitation of our study may be the assumption made for the primary outcome. We chose to use the results of Gharib and Rabab [23] to calculate the power of the study because the reported mean walking speeds are close to the walking speeds observed in our patients. In their study, which investigated the effect of isokinetic strengthening of the knee and ankle muscles, the difference in walking speed after treatment was 0.09 m/s. This value could be considered as not clinically relevant. However, in a recent study, Lewek and Sykes [25] showed that Minimal Detectable Change (MDC) is related to walking speed. For post-stroke patients walking slowly (< 0.4 m/s), the MDC could be 0.10 m/s, which is very close to the value we chose. Another limitation could be the use of an isokinetic dynamometer. We do not think that this is a problem, as this type of device is now readily available in rehabilitation departments. Finally, it is not possible to propose a double-blind protocol. Indeed, the patient and the therapist cannot be blind to the procedure. This is very often the case in studies evaluating rehabilitation programmes. To limit the bias, however, the evaluators are blind and do not know to which group the patient belongs.

This research should also contribute to the optimisation of physical medicine and rehabilitation practices by including, in a more systematic way, an objective evaluation of the rehabilitative benefits: instrumental evaluation of walking and evaluation of the strength of the muscles involved in this activity. The use of the isokinetic dynamometer as an associated technique integrated into the conventional rehabilitation protocol should increase the rehabilitative gain in central neurological pathologies.

### Trial status

- Version 2: February 2, 2021
- Overall status: recruitment in progress.
- Study start: May 20, 2021
- Recruitment end date: November 20, 2022
- Study end: November 20, 2023

## Data Availability

Once cleaned, the data are available for analysis and the results returned to the coordinating investigator for publication. Any written or oral communication of study results must be approved by the coordinating investigator.

## Abbreviations

10MWT: 10-m walking test
A-ROM: Active range of motion
BDAE: Boston Diagnostic Aphasia Examination
BI: Barthel index
CG: Control group
CPM: Continuous passive mobilization
eCRF: Electronic case report form
EG: Experimental group
FAC: Functional Ambulation Classification
ICC: Intra-class correlation coefficient
MAS: Modified Ashworth Scale
MRC: Medical Research Council testing
NIHSS: National Institute of Health Stroke Score
P-ROM: Passive range of motion
RCT: Randomised controlled trial
SIPSO: Subjective Index of Physical and Social Outcome
T12M: Time12months
T6M: Time6months
VAS: Visual analogue scale
WAKE: Walking Ankle isoKinetic Exercise
